# Patient-Reported Outcomes After Lumbar Fusion Using Transforaminal vs. Lateral Lumbar Interbody Fusion Across BMI Categories in Low-Grade Spondylolisthesis

**DOI:** 10.7759/cureus.86582

**Published:** 2025-06-23

**Authors:** Rafael A Garcia, Kari Odland, Jonathan Sembrano

**Affiliations:** 1 Orthopedic Surgery, University of Minnesota, Minneapolis, USA

**Keywords:** body mass index, lateral lumbar interbody fusion, lumbar spondylolisthesis, patient-reported outcomes, transforaminal lumbar interbody fusion

## Abstract

Background: Lumbar spondylolisthesis often causes pain and disability, requiring surgical intervention. While prior studies explore the impact of body mass index (BMI) on outcomes following lumbar fusion, the effect of different surgical approaches, specifically transforaminal lumbar interbody fusion (TLIF) and lateral lumbar interbody fusion (LLIF), across BMI categories remains unclear. This study evaluates patient-reported outcomes (PROs) following TLIF and LLIF in low-grade degenerative and isthmic spondylolisthesis, focusing on BMI stratification.

Methods: This study retrospectively analyzes patients with low-grade degenerative and isthmic spondylolisthesis who underwent lumbar fusion between 2010 and 2023. Patients were stratified by BMI into the following four groups: non-obese (<30), class I (30-34.9), class II (35-39.9), and class III (≥40). It assesses PROs using the Oswestry Disability Index (ODI) and visual analog scale (VAS) for pain at baseline and 12 months, comparing the achievement of minimal clinically important difference (MCID) between TLIF and LLIF.

Results: This analysis included 72 patients showing significant improvements in ODI (mean change: 17.0; p<0.001) and VAS (mean change: 2.3; p<0.001) scores across all BMI categories at 12 months. TLIF and LLIF achieve similar rates of MCID, with no statistically significant differences between the surgical approaches (p=0.72). Non-obese and class II patients maintain sustained improvements.

Conclusion: Lumbar fusion using both open and minimally invasive (MIS) TLIF and LLIF leads to significant disability reduction across BMI categories, indicating that obesity does not contraindicate lumbar fusion. Surgical approach selection should focus on individual patient factors rather than BMI alone. Prospective studies with extended follow-up will further clarify the long-term impact of these approaches.

## Introduction

Lumbar spondylolisthesis, characterized by vertebral slippage, can lead to chronic back pain, reduced functionality, and disability, often requiring surgical intervention [1]. As obesity continues to rise, it is critical to understand how different BMI categories affect outcomes following lumbar fusion [2]. While obesity has been associated with heightened perioperative risks, its effects on recovery and functional outcomes after lumbar fusion remain an area of ongoing investigation [3]. A recent study by our group examined the influence of BMI on patient-reported outcomes (PROs) following lumbar fusion in isthmic and degenerative spondylolisthesis patients. The findings revealed significant disability reduction across all BMI categories, with sustained improvements in non-obese and class II obesity patients (unpublished data, University of Minnesota).

Building on these findings, this follow-up study aimed to explore whether the surgical approach affects outcomes across different BMI categories. While prior research has focused primarily on anterior lumbar interbody fusion (ALIF), posterior lumbar interbody fusion (PLIF), and posterolateral fusion (PLF), this study specifically investigates open lateral lumbar interbody fusion (LLIF) and transforaminal lumbar interbody fusion (TLIF) in low-grade degenerative and isthmic spondylolisthesis patients [1].

## Materials and methods

Case ascertainment

This study utilizes the same institutional cohort and methodological framework previously described in detail by Garcia et al. [4]. Data for this study originate from patients with diagnosed low-grade degenerative and isthmic spondylolisthesis at a single institution between 2010 and 2023. Patients eligible for analysis (n=271) were categorized into National Institutes of Health (NIH) BMI classes by preoperative weight. Of these, 199 were excluded because they did not undergo lumbar spinal fusion or were not diagnosed with spondylolisthesis. The BMI classes were defined as <30 kg/m^2^ non-obese, 30.0-34.9 kg/m^2^ obesity class I, 35.0-39.9 kg/m^2^ obesity class II, and ≥40.0 kg/m^2^ obesity class III.

Patient population

Eligibility criteria included the following: adult patients (≥18 years) with a confirmed diagnosis of lumbar spondylolisthesis (L1-S1) due to degeneration and/or pars defect. Patients were classified using Meyerding's classification (grades 1 or 2) and had undergone one or multiple levels of lumbar fusion utilizing open or minimally invasive transforaminal lumbar interbody fusion (TLIF) or lateral lumbar interbody fusion (LLIF). Exclusion criteria included patients with other spinal conditions requiring surgical intervention (e.g., scoliosis, trauma, iatrogenic conditions, or spondylosis) or those who had not undergone lumbar spinal fusion (i.e., decompression-only surgery).

Follow-up

Outcome measures of pain and disability were collected at patient follow-up visits preoperatively and at 12 months. Back pain was assessed using a 0-10 visual analog scale (VAS). Dysfunction related to pain was assessed using the Oswestry Disability Index (ODI), a validated and commonly used questionnaire addressing dysfunction due to back pain [5,6]. The minimum clinically important difference (MCID) represents the smallest change in a patient-reported outcome measure that is of genuine clinical value to patients [7], and the values used for MCID in the current study were 13 for ODI and 2 for VAS [4,8].

Statistical analysis

All analyses focused on patients diagnosed with low-grade degenerative and isthmic spondylolisthesis who underwent spinal fusion. Patient-reported outcomes and changes from baseline were compared using the BMI class and surgical approach, using a paired Student's t-test and a one-way analysis of variance (ANOVA) with post-hoc Bonferroni pairwise comparisons at each time interval. Previously published distribution-based methods were applied to examine the achievement of the MCID. All statistical analyses were performed using SPSS version 29.0 (Armonk, NY: IBM Corp.). P-values <0.05 were considered statistically significant.

## Results

Baseline characteristics

**Figure 1:**
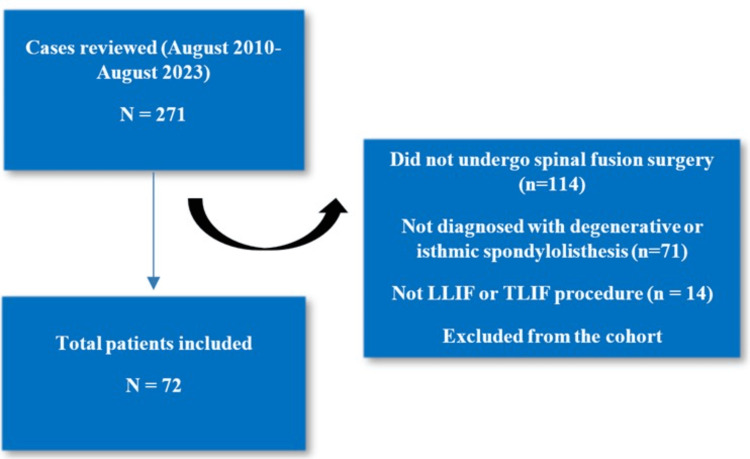
Methodology CONSORT diagram. TLIF: transforaminal lumbar interbody fusion; LLIF: lateral lumbar interbody fusion; CONSORT: Consolidated Standards of Reporting Trials; N: total number

A total of 271 patients were eligible for analysis, but 72 patients (42 females and 30 males) met inclusion criteria and were included in the study (Figure 1 and Tables 1-3). The mean BMI was 31.02 (±6.84) (Tables 1-3). Among the respective BMI groups, patients with a <30 BMI comprised the majority of the cohort 30/72 (41.7%), compared to the 30-34.9 BMI group 23/72 (31.4%), the 35-39.9 BMI group 14/72 (19.4%), and the >40 BMI group 5/72 (6.9%). Two patients reported prior lumbar fusion history, and six patients reported a history of degenerative disc disease (Tables 1-3). The types of surgeries performed included 37 (51.4%) TLIFs and 35 (48.6%) LLIFs (Tables 1-3). Meyerding’s spondylolisthesis classification was utilized to grade the spondylolisthesis, which included 47 (72.3%) grade 1 and 18 (27.7%) grade 2 (Tables 1-3). Patients reported a mean baseline pain (VAS) score of 5.83 (±2.73), with five patients reporting a pain score of 0/10 (none), nine patients reporting a pain score of 1-3/10 (mild), 21 patients reporting a pain score of 4-6/10 (moderate), and 37 patients reporting a pain score of 7-10/10 (severe) (Tables 1-3). Patients reported a mean baseline disability (ODI) score of 47.44 (±15.34) with four patients reporting a disability score between 0 and 20 (minimal), 16 patients reporting a disability score of 21-40 (moderate), 40 patients reporting a disability score of 41-60 (severe), 12 patients reporting a disability score of 61-80 (crippled), and 0 patients reporting a disability score of 81-100 (bedbound) (Tables 1-3).

**Table 1:** Overall cohort characteristics. VAS: visual analog scale; ODI: Oswestry Disability Index

Variables	Frequency	n	%
Demographics	Males	30	41.7
	Females	42	58.3
	BMI <30	30	41.7
	BMI 30-34.9	23	31.9
	BMI 35-39.9	14	19.4
	BMI ≥40	5	6.9
VAS	0 None	5	6.9
	1-3 Mild	9	12.5
	4-6 Moderate	21	29.2
	7-10 Severe	37	51.4
ODI	0-20 Minimal	4	5.6
	21-40 Moderate	16	22.2
	41-60 Severe	40	55.6
	61-80 Crippled	12	16.7
	81-100 Bedbound	0	0
Total		72	100.0

**Table 2:** Mean values for overall cohort characteristics. VAS: visual analog scale; ODI: Oswestry Disability Index

Variables	Mean±SD
Age	65.1±13.5
BMI	31.02±6.8
VAS	5.8±2.7
ODI	47.4±15.3

**Table 3:** Overall cohort diagnostic and surgical characteristics. TLIF: transforaminal lumbar interbody fusion; LLIF: lateral lumbar interbody fusion

Variables	Frequency	n	%
Prior lumbar fusion	Yes	2	2.8
	No	70	97.2
Degenerative disk disease	Yes	6	8.3
	No	66	97.2
Type of surgery	TLIF	37	51.4
	LLIF	35	48.6
Segment level	L3-4	9	12.5
	L4-5	40	55.6
	L5-S1	9	12.5
	Multilevel	14	19.4
Spondylolisthesis grade	Grade 1	47	72.3
	Grade 2	18	27.7
Spondylolisthesis type	Degenerative	51	70.8
	Isthmic	21	29.2
Total		72	100.0

Tables 4-6 compare the baseline characteristics of patients undergoing TLIF and LLIF. The TLIF cohort includes 17 females (45.9%) and 20 males (54.1%), while the LLIF cohort comprises 25 females (71.4%) and 10 males (28.6%). The mean age of patients in the TLIF cohort is slightly lower at 62.88 years (±16.27), compared to 67.32 years (±9.92) in the LLIF cohort. The mean BMI is also lower in the TLIF group, with an average of 28.55 (±6.75), while the LLIF group averages 33.63 (±5.97), reflecting a higher proportion of obese patients in the LLIF cohort. Regarding prior lumbar fusion history, one patient in each cohort had a history of lumbar fusion (TLIF: 2.7%; LLIF: 2.9%). The presence of degenerative disk disease is more common in the TLIF cohort (10.8%) than in the LLIF cohort (5.7%). Segmental levels treated also vary between the groups, with TLIF predominantly targeting L4-5 (48.6%), while LLIF is primarily performed at L4-5 (62.9%) and L3-4 (22.9%). Grade 1 spondylolisthesis is more common in the LLIF group (84.4%) compared to the TLIF group (60.6%), which shows a higher rate of grade 2 cases (39.4%). Baseline pain and disability levels are similar between the groups, with TLIF patients reporting a mean VAS score of 5.77 (±2.87) and LLIF patients reporting 5.89 (±2.62). Baseline ODI scores are slightly lower in the TLIF cohort, averaging 42.46 (±18.74), compared to 49.52 (±15.93) in the LLIF cohort.

**Table 4:** Characteristics between TLIF and LLIF cohorts. TLIF: transforaminal lumbar interbody fusion; LLIF: lateral lumbar interbody fusion; VAS: visual analog scale; ODI: Oswestry Disability Index

Variables	Frequency	TLIF (n)	LLIF (n)
Demographics	Males	20	10
	Females	17	25
	BMI <30	21	9
	BMI 30-34.9	9	14
	BMI 35-39.9	6	8
	BMI ≥40	1	4
VAS	0 None	3	2
	1-3 Mild	4	5
	4-6 Moderate	10	11
	7-10 Severe	20	17
ODI	0-20 Minimal	3	1
	21-40 Moderate	9	7
	41-60 Severe	20	20
	61-80 Crippled	5	7
	81-100 Bedbound	0	0
Total		37	35

**Table 5:** Mean values for characteristics between TLIF and LLIF cohorts. TLIF: transforaminal lumbar interbody fusion; LLIF: lateral lumbar interbody fusion; VAS: visual analog scale; ODI: Oswestry Disability Index

Variables		Mean±SD
TLIF	Age	62.88±16.27
	BMI	28.55±6.75
	VAS	5.77±2.87
	ODI	42.46±18.74
LLIF	Age	67.32±9.92
	BMI	33.63±5.97
	VAS	5.89±2.62
	ODI	49.52±15.93

**Table 6:** Diagnostic and surgical characteristics between TLIF and LLIF cohorts. TLIF: transforaminal lumbar interbody fusion; LLIF: lateral lumbar interbody fusion

Variables		TLIF Frequency (n)	LLIF Frequency (n)	p-Value
Prior lumbar fusion	Yes	1	1	0.96
	No	36	34	
Degenerative disk disease	Yes	4	2	0.43
	No	33	33	
Segment level	L3-4	1	8	0.003
	L4-5	18	22	
	L5-S1	8	1	
	Multilevel	10	4	
Spondylolisthesis grade	Grade 1	20	27	0.004
	Grade 2	13	5	
Spondylolisthesis type	Degenerative	24	27	0.25
	Isthmic	13	8	
Total		37	35	

Primary outcomes for all surgical approaches and BMI categories

Overall, the mean VAS pain score improved from 5.9 at baseline to 3.6 at 12 months for the cohort, signaling a 2.3-point improvement (p<0.001). The mean ODI score improved from 46.1 at baseline to 29.1 at 12 months, signaling a 17.0-point improvement (p≤.001) (Table 2). The VAS mean change from baseline to 12-month follow-up for each surgical approach is not statistically significant (p=0.11). The TLIF cohort met MCID. The ODI mean change from baseline to 12-month follow-up for each surgical approach is not statistically significant (p=0.72). Both the TLIF and LLIF cohorts meet the MCID.

Primary outcomes for <30 BMI cohort for all surgical approaches

**Figure 2:**
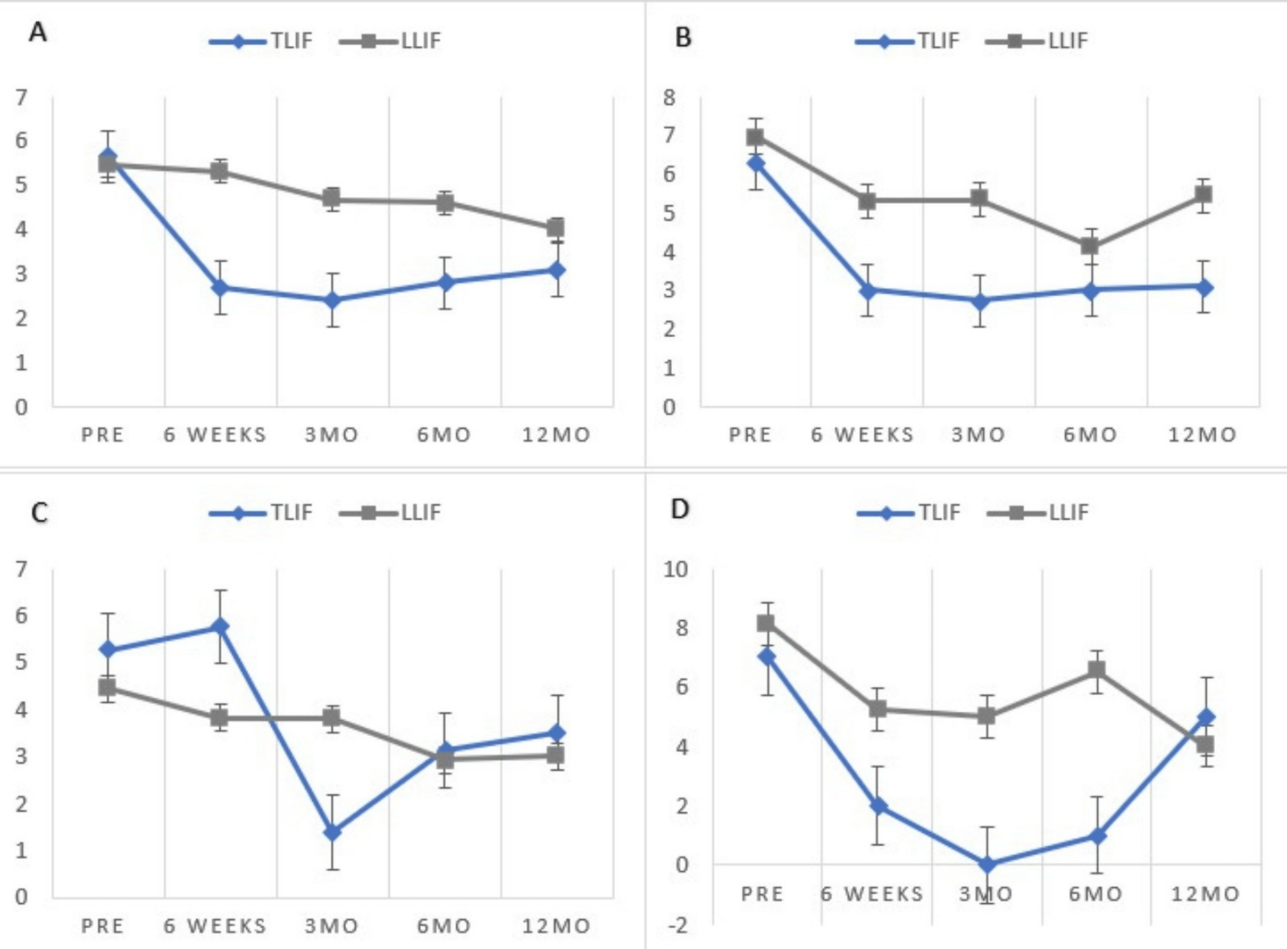
Patient-reported outcome improvements by BMI group - TLIF cohort. TLIF: transforaminal lumbar interbody fusion; LLIF: lateral lumbar interbody fusion; MO: months; PRE: preoperative

**Figure 3:**
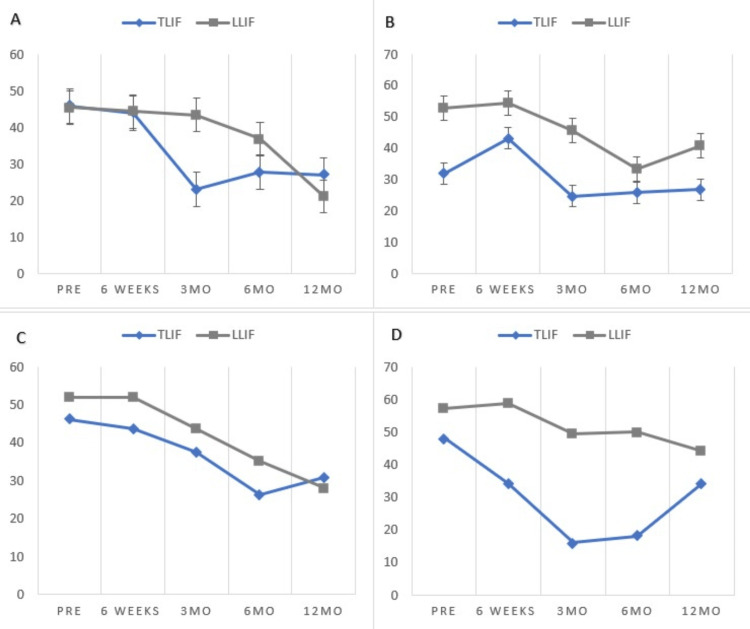
Patient-reported outcome improvements by BMI Group - LLIF cohort. TLIF: transforaminal lumbar interbody fusion; LLIF: lateral lumbar interbody fusion; MO: months; PRE: preoperative

Non-obese patients who underwent a TLIF have statistically significant VAS change from preoperative to 12-month postoperative follow-up (p=0.01), and the mean change met MCID (3.05). ODI change is statistically significant from preoperative to 12-month postoperative follow-up (p<0.001), and the mean change meets MCID (19.75). Non-obese patients who underwent an LLIF did not have a statistically significant VAS change from preoperative to 12-month postoperative follow-up (p=0.74), and MCID was not met (0.67). ODI change is statistically significant from preoperative to 12-month postoperative follow-up (p=0.27), and MCID is met (Figure 2, panel A and Figure 3, panel A).

Primary outcomes for the 30-34.9 BMI cohort for all surgical approaches

Class I obese patients who underwent a TLIF with a BMI 30-34.9 do not have a statistically significant VAS change from preoperative to 12-month postoperative follow-up (p=0.051), although the mean change met MCID (3.17). ODI change is not statistically significant from preoperative to 12-month postoperative follow-up (p=0.613), and the mean change does not meet MCID (5.06). Class I obese patients who underwent an LLIF do not have a statistically significant VAS change from preoperative to 12-month postoperative follow-up (p=0.132), and the mean change does not meet MCID (1.39). ODI change was statistically significant from preoperative to 12-month postoperative follow-up (p=0.035), but the mean change did not meet MCID (10.40) (Figure 2, panel b and Figure 3, panel b).

Primary outcomes for 35-39.9 BMI cohort for all surgical approaches

Class II obese patients who underwent a TLIF have a statistically significant VAS change from preoperative to 12-month postoperative follow-up (p=0.049), and the mean change met MCID (3.25). ODI change is statistically significant from preoperative to 12-month postoperative follow-up (p=0.003), and the mean change meets MCID (17.86). Class II obese patients who underwent an LLIF do not have a statistically significant VAS change from preoperative to 12-month postoperative follow-up (p=0.133), and the mean change does not meet MCID (1.39). ODI change is statistically significant from preoperative to 12-month postoperative follow-up (p=0.004), and the mean change meets MCID (19.36) (Figure 2, panel c and Figure 3, panel c).

Class III obese patients who underwent an LLIF do not have a statistically significant VAS change from preoperative to 12-month postoperative follow-up (p=0.204), and the mean change meets MCID (4.33). ODI change is not statistically significant from preoperative to 12-month postoperative follow-up (p=0.215), and the mean change meets MCID (11.59) VAS and ODI t-tests for class III patients who underwent TLIF could not be reported because the number of patients in the cohort (Figure 2, panel d and Figure 3, panel d).

## Discussion

This study provides insights into how different surgical approaches, open TLIF and LLIF, impact outcomes in lumbar spondylolisthesis across BMI categories. Building on our prior study, which found meaningful improvements in disability across all BMI classes, this analysis aimed to determine if surgical technique modulates these outcomes [8]. The findings suggest that while significant disability reduction and pain improvement were observed across BMI groups, there were no statistically significant differences in outcomes between the TLIF and LLIF techniques in terms of achieving minimal clinically important difference (MCID) at 12 months (p=0.72).

The comparable effectiveness of TLIF and LLIF aligns with several studies that report similar functional outcomes for both techniques, irrespective of BMI. Findings are consistent with a two-year comparative study, which demonstrated no significant differences in pain relief, disability reduction, or overall patient satisfaction between minimally invasive TLIF (MIS-TLIF) and LLIF (also known as XLIF or extreme lateral interbody fusion [9]. Highlighting that while XLIF provided reduced blood loss and shorter operative times, clinical outcomes in pain and disability were equivalent to those achieved with TLIF at the two-year mark, supporting the notion that both approaches are viable options for spondylolisthesis management across BMI categories [9]. Lee et al. reported similar improvements in patient-reported outcomes, reinforcing that factors such as patient anatomy and clinical context, rather than BMI alone, should guide surgical decision-making [10]. The comparable outcomes observed between TLIF and LLIF may be due to their shared biomechanical objectives. Both achieve neural decompression and segmental stabilization, critical for functional recovery in lumbar spondylolisthesis. TLIF provides direct decompression and posterior column support, while LLIF offers greater restoration of disc height and sagittal balance through indirect decompression, which may explain the consistent results across BMI groups [11].

This study’s results align with existing literature that reports equivalent functional outcomes between TLIF and LLIF [1], suggesting that the mechanism of decompression, whether direct or indirect, achieves the same clinical endpoints for low-grade degenerative and isthmic spondylolisthesis [9,10]. The perioperative benefits observed in LLIF, such as reduced blood loss and shorter operative times, are particularly relevant for obese patients, but the lack of significant differences in long-term outcomes supports the notion that stabilization and neural decompression are the primary drivers of clinical success. These findings suggest that, beyond BMI, the surgical technique should be chosen based on specific patient anatomy, the severity of stenosis, and the surgeon's expertise, aligning with broader surgical principles found in related studies. Future studies should focus on longer-term outcomes to assess sustained improvements, particularly in class III patients, where outcomes plateau at 12 months. Randomized controlled trials are needed to compare TLIF and LLIF outcomes across BMI categories definitively, considering both clinical and radiographic metrics to refine surgical decision-making.

As a retrospective analysis, this introduces inherent biases, limiting control over confounding variables. Smaller sample size for class III obesity reduces the statistical power to detect differences across surgical approaches. Twelve-month follow-up may not capture long-term outcomes, particularly for higher BMI groups where initial gains plateaued. The variability in surgical technique and lack of radiographic assessments may influence outcomes, as this study focuses solely on patient-reported outcomes. Lastly, uncontrolled factors, such as comorbidities and socioeconomic status, could contribute to recovery variability.

## Conclusions

Lumbar fusion achieves meaningful improvements in pain and disability across BMI categories, regardless of the surgical approach used. Both TLIF and LLIF are effective in managing low-grade degenerative and isthmic spondylolisthesis, with no significant differences in patient-reported outcomes at one-year follow-up. These findings build upon our previous work, confirming that obesity should not be considered a contraindication for lumbar fusion, as well-selected patients can achieve significant disability reduction across BMI groups.
